# Evidence From a Systematic Review and Meta-Analysis: Classical Impaired Glucose Tolerance Should Be Divided Into Subgroups of Isolated Impaired Glucose Tolerance and Impaired Glucose Tolerance Combined With Impaired Fasting Glucose, According to the Risk of Progression to Diabetes

**DOI:** 10.3389/fendo.2022.835460

**Published:** 2022-02-18

**Authors:** Yupu Liu, Juan Li, Yuchao Wu, Han Zhang, Qingguo Lv, Yuwei Zhang, Xiaofeng Zheng, Nanwei Tong

**Affiliations:** ^1^ Department of Endocrinology and Metabolism, West China Hospital of Sichuan University, Chengdu, China; ^2^ Laboratory of Diabetes and Islet Transplantation Research, Center for Diabetes and Metabolism Research, West China Hospital of Sichuan University, Chengdu, China; ^3^ Department of Endocrinology, Affiliated Hospital of Zunyi Medical University, Zunyi, China

**Keywords:** type 2 diabetes, prediabetes, classification, diagnostic criteria, review systematic

## Abstract

**Background:**

The American Diabetes Association (ADA) 2003 diagnostic criteria divide impaired glucose tolerance (IGT) into isolated impaired glucose tolerance with normal fasting glucose (I-IGT, IGT+NFG) and impaired glucose tolerance combined with impaired fasting glucose (IGT+IFG), while the World Health Organization (WHO) 1999 criteria do not. The aim of this meta-analysis was to evaluate whether IGT should be divided into I-IGT (IGT+NFG) or IGT+IFG according to their risk of progression to type 2 diabetes.

**Methods:**

The MEDLINE and EMBASE were searched to identify prospective cohort studies published in English prior to April 18, 2020. Review Manager 5.3 was used to calculate the pooled risk ratios (RRs) and 95% confidence intervals (CIs) as summary statistics for each included study.

**Results:**

Sixteen eligible studies (*n* = 147,006) were included in the analysis. The subsequent incidence of type 2 diabetes was lower in the I-IGT (IGT+NFG) group than in the IGT+IFG group (0.45 [95% CI 0.37, 0.55] according to WHO 1999 criteria and 0.59 [95% CI 0.54, 0.66] according to ADA 2003 criteria). It was higher in the I-IFG, I-IGT (IGT+NFG), and IGT+IFG groups than in the normoglycemic group (95% CI of 5.53 [3.78, 8.08], 5.21 [3.70, 7.34], and 11.87 [7.33, 19.20] according to the WHO 1999 criteria and 95% CI of 2.66 [2.00, 3.54], 3.34 [2.81, 3.97], and 6.10 [4.72, 7.88] according to the ADA 2003 criteria). In general, the incidence of diabetes in the IGT+IFG group was the highest in the prediabetic population.

**Conclusions:**

The present meta-analysis suggested that the established WHO diagnostic criteria for IGT should be revised to separately identify individuals with IGT+NFG or IGT+IFG.

## Introduction

With the development of society and changes in lifestyle, the global incidence of diabetes and expenditure on the disease are constantly increasing. The global estimate of the number of adults with diabetes was 425 million in 2017, and this number is expected to rise beyond 629 million by 2045. Furthermore, USD 727 billion was spent on diabetes in 2017 (1). Thus, diabetes places a significant burden on both the health of the population and economies. Prediabetes is defined as an intermediate metabolic state between normal and diabetic glucose homeostasis and is characterized by insulin resistance and beta-cell dysfunction (2, 3). There are 352.1 million adults of 20–79 years worldwide who are thought to have impaired glucose tolerance (IGT), with or without impaired fasting glucose (IFG), or 1 in 14 adults (1). Prediabetes can be reversed by medication and lifestyle interventions; therefore, early detection and intervention for prediabetes can reduce the incidence of diabetes and the related cardiovascular disease and death (4).

The World Health Organization (WHO) 1999 guidelines for the definition of prediabetes state that prediabetes can be diagnosed on the basis of either isolated impaired fasting glucose (I-IFG: 6.1 mmol/L ≤FPG <7.0 mmol/L) or IGT (5) (FPG <7.0 mmol/L and 7.8 mmol/L ≤OGTT 2-h plasma glucose < 1.1 mmol/L), meaning that they do not distinguish between individuals with normal fasting glucose (NFG) and those with IFG when their 2-h plasma glucose during an OGTT is ≥7.8 mmol/L (140 mg/dl) and <11.1 mmol/L (200 mg/dl). In contrast, three different groups of prediabetic patients are defined in the American Diabetes Association (ADA) 2003 guidelines: those with isolated impaired fasting glucose (I-IFG), those with isolated impaired glucose tolerance with normal fasting glucose (I-IGT, IGT+NFG), and those with impaired glucose tolerance combined with impaired fasting glucose (IGT+IFG) (6). The ADA 2003 criteria also divide individuals with impaired glucose tolerance into those with normal fasting glucose (IGT+NFG) and those with impaired fasting glucose (IGT+IFG), also called I-IGT and IGT+IFG, respectively. Thus, there are some differences between the WHO and ADA definition of prediabetes, and it is unclear whether the WHO definition of IGT should be refined to specify whether patients have I-IGT or IGT+IFG.

People with prediabetes are at high risk of developing to type 2 diabetes (7–9). Moreover, many studies have reported that people with IGT (10–12) or IFG (13–15) are at higher risk of type 2 diabetes and its complications. However, few studies have clearly distinguished subjects with I-IFG, I-IGT (IGT+NFG), or IGT+IFG or reported the risks of progression to type 2 diabetes for these subgroups of people with prediabetes. Therefore, we aimed to perform a systematic review and meta-analysis of the progression to type 2 diabetes for these different prediabetic groups, to explore whether population with WHO-defined IGT should be divided into those with I-IGT (IGT+NFG) or IGT+IFG.

## Methods

This systematic review and meta-analysis was conducted in accordance with the meta-analysis of observational studies in epidemiology (MOOSE) (16).

### Information and Search Strategy

We performed a systematic literature search of Medline and EMBASE from inception to April 18, 2020. MeSH combined with free words terms about “prediabetes or impaired fasting glucose or impaired glucose tolerance” and “follow-up or progress or prediction” were used to identify relevant articles (see **Supplementary Material 1**, which illustrates the search strategies). We restricted the search to articles in English.

### Study Eligibility and Selection

The inclusion criteria were as follows: (a) study design: prospective cohort studies with oral glucose tolerance test (OGTT) performed at baseline; fasting plasma glucose (FPG) and 2-h postprandial plasma glucose (2hPG) were recorded, and other risk factors on progression to type 2 diabetes were not considered; (b) follow-up period: at least 3 years; (c) population: prediabetic individuals who were clearly divided into those with I-IFG, I-IGT (IGT+NFG), or IGT+IFG and a normoglycemic population, all individuals were derived from the general population; (d) outcome: progression to type 2 diabetes; (e) publication type: primary studies; and (f) language: English. Exclusion criteria were as follows: case-control studies; meta-analysis or systematic studies; studies without data on a normoglycemic population; intervention studies (medication, lifestyle, or physical activity); letters to the editor; consensus conference reports; editorials; and practice guidelines. The identified records were managed by EndNote reference management software version X7 (Thomson Reuters, Columbus, OH, USA).

Prediabetes was defined as impaired fasting glucose or impaired glucose tolerance. Using fasting plasma glucose and 2-h plasma glucose during an OGTT, three different subgroups of prediabetes can be defined: I-IFG, I-IGT (IGT+NFG), and IGT+IFG. The I-IFG group was defined as having impaired fasting glucose and normal glucose tolerance; the I-IGT (IGT+NFG) group was defined as having impaired glucose tolerance and normal fasting glucose; and the IGT+IFG group was defined as having impaired fasting glucose combined with impaired glucose tolerance. Therefore, we divided individuals with prediabetes into three groups according to the WHO 1999 plasma glucose cutpoint values and 2003 ADA Prediabetes subtype: WHO I-IFG: fasting blood glucose is greater than or equal to 6.1 mmol/L (110 mg/dl) and less than 7.0 mmol/L (126 mg/dl), and OGTT 2-h plasma glucose is less than 7.8 mmol/L (140 mg/dl); WHO I-IGT (IGT+NFG): fasting blood glucose is less than 6.1 mmol/L (110 mg/dl), and OGTT 2-h plasma glucose is greater than or equal to 7.8 mmol/L (140 mg/d) and less than 11.1 mmol/L (200 mg/dl); and WHO IGT+IFG: fasting blood glucose is greater than or equal to 6.1 mmol/L (110 mg/dl) and less than 7.0 mmol/L (126 mg/dl), and OGTT 2-h plasma glucose is greater than or equal to 7.8 mmol/L (140 mg/dl) and less than 11.1 mmol/L (200 mg/dl) (5).

We also divided prediabetes into three groups according to the ADA 2003 criteria: ADA I-IFG: fasting blood glucose is greater than or equal to 5.6 mmol/L (100 mg/dl) and less than 7.0 mmol/L (126 mg/dl), and OGTT 2-h plasma glucose is less than 7.8 mmol/L (140 mg/dl); ADA I-IGT (IGT+NFG): fasting blood glucose is less than 5.6 mmol/L (100 mg/dl), and OGTT 2-h plasma glucose is greater than or equal to 7.8 mmol/L (140 mg/dl) and less than 11.1 mmol/L (200 mg/dl); and ADA IGT+IFG: fasting blood glucose is greater than or equal to 5.6 mmol/L (100 mg/dl) and less than 7.0 mmol/L (126 mg/dl), and OGTT 2-h plasma glucose is greater than or equal to 7.8 mmol/L (140 mg/dl) and less than 11.1 mmol/L (200 mg/dl) (6).

### Study Process

Two reviewers (YL and JL) independently screened the titles and abstracts of the articles, and full-text articles of potentially suitable studies were obtained. Disagreements were resolved by consensus or by seeking the opinion of a third reviewer. The two primary reviewers also independently extracted data regarding the following characteristics of the included studies: study group, first author, publication year, diagnostic criteria, study location, duration of follow-up, sample size, and the number of outcomes. Extracted data were entered into a standardized Microsoft Office Word 2003 template (Microsoft Corporation, Redmond, WA, USA) file.

### Quality Assessment

We used the Newcastle-Ottawa quality assessment scale for quality assessment of the included studies (17). Two authors (YW and YL) independently assessed the quality of each included study on the basis of selection method (four items), comparability (one item), and outcome (three items). We graded the quality as good (≥7 stars), fair (4–6 stars), and poor (<4 stars) in this meta-analysis (18, 19). Publication bias was accessed and illustrated by Egger’s plot.

### Statistical Analysis

We used risk ratios (RRs, Mantel-Haenszel method) with 95% confidence intervals (CIs) as summary statistics for each included study; sensitivity analyses were conducted by using alternative effect measures (RR). These were calculated from the number of events and the total population size at baseline for each study. We used the *I* (2) statistic to test statistical heterogeneity, which is a quantitative measure of inconsistency among studies. Analyses with an *I* (2) statistic ≤50% were considered to have no significant heterogeneity and analyses with an *I* (2) statistic >50% were considered to have significant heterogeneity, a random-effect model was used for analyses (20).

A subgroup analysis was performed based on the follow-up time and study location to explore whether it would affect the final outcome. In addition, the risk of progression to type 2 diabetes in different prediabetes subgroups were analyzed according to glucose tolerance status under WHO 1999 and ADA 2003 diagnostic criteria of plasma glucose level, respectively.

We repartitioned the included studies into WHO and ADA categories to compare the risk of progression with type 2 diabetes in the I-IGT (IGT+NFG) group with that in the IGT+IFG group. We also compared the risk of development of type 2 diabetes in the I-IFG, I-IGT (IGT+NFG), and IGT+IFG groups with that for the normoglycemic group.

Analyses were performed with RevMan 5.3 (Cochrane Collaboration, Copenhagen, Denmark) and Stata 12.0 (StataCorp, LP, College Station, TX, USA).

## Results

### Study Selection and Characteristics

**Figure 1:**
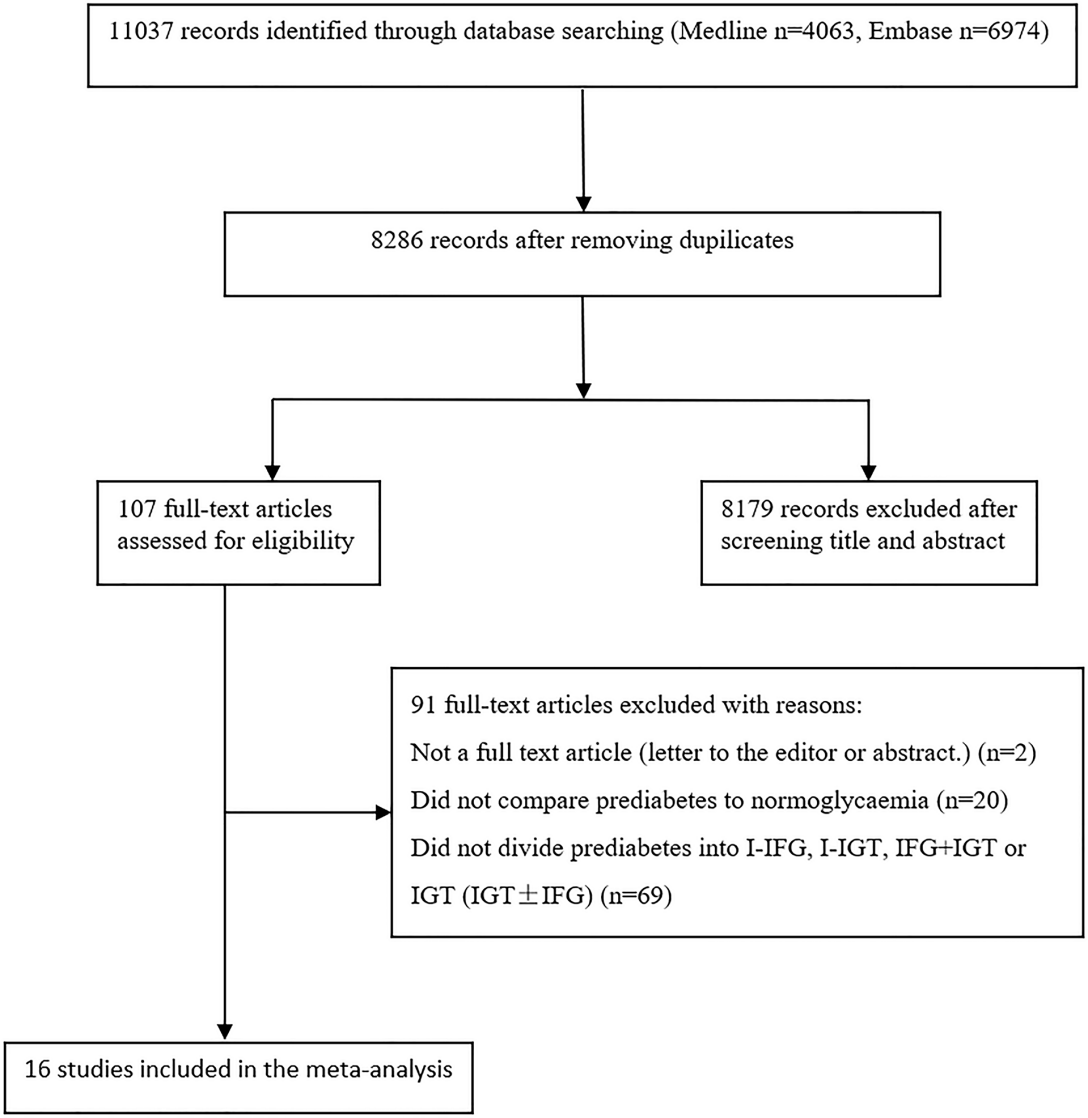
Study selection flow chart.

**Figure 1** is a flow chart describing study selection. The database searches identified 11,037 records. After removing duplicates, 8,286 records remained. A total of 107 records were selected for full-text assessment after screening the titles and abstracts. After full-text screening, we identified 16 eligible studies for the meta-analysis (21–33).

The duration of follow-up varied from 3 to 12 years, with a median duration of 5.6 years. All individuals were derived from the general population. However, the fasting plasma glucose of the subjects in the Li 2003 study (23) was 5.6–7.0 mmol/L and the 2-h postprandial capillary blood glucose was ≥6.67 mmol/L in the subjects studied by Wang (25). There was a broad representation of ethnicities, including populations from China, America, Finland, the Netherlands, Korea, and India.

There were 9 studies that used the WHO 1999 definition of impaired fasting glucose (6.1–6.9 mmol/L) (21–24, 26, 28, 29, 31, 34), 5 studies that used the ADA 2003 criteria (5.6–6.9 mmol/L) (30, 32, 33, 35, 36), and 2 study that used both definitions (25, 27) (see **Tables 1**, **2**). We analyzed 26,159 individuals defined using the WHO 1999 plasma glucose range and 120,847 individuals defined using the ADA 2003 plasma glucose range.

**Table 1:** Characteristics of the included studies according to the WHO 1999 classification criteria.

Author (year)	Study group	Study duration (year)	Study location	Baseline (*n*)		Baseline (*n*)		Baseline (*n*)		Baseline (*n*)		Effects estimator	Adjusted covariate
				WHO NFG+NGT		WHO I-IFG		WHO I-IGT		WHO IGT+IFG			
				Total number of population	Number of progression to T2DM	Total number of population	Number of progression to T2DM	Total number of population	Number of progression to T2DM	Total number of population	Number of progression to T2DM		
Gabir (2000) (21)	Pima	5	North America	3,499	126	93	29	537	107	126	52	–	–
Vegt (2001) (22)	Hoorn	6.4	European	1,125	51	106	35	80	27	31	20	OR	Adjusted for follow-up duration, age, and sex.
Li (2003) (23)	Kinmen	5	Asia	435	38	42	16	118	33	49	20	HR	Adjusted for gender, age, and obesity.
Qiao (2003) (24)	FINMONICA	10	European	2,129	44	104	5	322	34	38	17	HR	Adjusted for age, sex, BMI, HDL, and WHR.
Wang (2004) (25)	–	5	Asia	358	51	112	28	95	31	62	36	OR	Grouped by sex and adjusted for age, smoking, SBP, BMI, family history of diabetes, total cholesterol, and triglycerides.
Valdes (2007) (26)	Asturias	6	Oceania	510	16	32	7	68	9	20	12	OR	Adjusted for age and sex.
Jia (2007) (27)	–	3	Asia	2,260	45	39	12	284	51	40	22	–	–
Harati (2009) (28)	TLGS	6	Asia	3,216	94	60	12	442	85	77	46	OR	Adjusted for age, sex, education level, family history of diabetes, hypertension, BMI, Abdominal obesity, triglyceride, and HDL.
Engberg (2009) (29)	Inter 99 Study	5	European	3,187	44	359	45	354	66	131	52	–	–
Qian (2012) (31)	–	5	Asia	843	59	46	17	120	49	33	17	–	–
Liu (2019) (34)	Reaction study	3	Asia	2,833	188	640	144	790	129	314	95	–	–

NFG+NGT, normal fasting glucose combined with normal glucose tolerance; I-IFG, isolated impaired fasting glucose; I-IGT, isolated impaired glucose tolerance; IGT+IFG, impaired fasting glucose combined with impaired glucose tolerance; T2DM, type 2 diabetes.

**Table 2:** Characteristics of the included studies according to the ADA 2003 classification criteria.

Author (year)	Study group	Study duration (year)	Study location	Baseline (*n*)		Baseline (*n*)		Baseline (*n*)		Baseline (*n*)		Effects estimator	Adjusted covariate
				ADA NFG+NGT		ADA I-IFG		ADA I-IGT		ADA IGT +IFG			
				Total number of population	Number of progression to T2DM	Total number of population	Number of progression to T2DM	Total number of population	Number of progression to T2DM	Total number of population	Number of progression to T2DM		
Wang (2004) (25)	–	5	Asia	209	26	261	53	48	13	109	54	OR	Grouped by sex and adjusted for age, smoking, SBP, BMI, family history of diabetes, total cholesterol, and triglycerides.
Wang (2010) (30)	SHS	7.8	North America	595	84	591	160	135	48	356	185	HR	Adjusted for age, sex, BMI, WC, albuminuria, smoking, family history of diabetes, and physical activity.
Anjana (2015) (32)	CURES	9.1	Asia	1,077	209	67	32	163	86	69	58	–	–
Han (2017) (33)	KoGES	12	Asia	5,633	657	199	81	1,512	624	198	138	HR	Adjusted for age and sex.
Jia (2007) (27)	–	3	Asia	2,079	31	247	26	215	28	109	45	–	–
Lu (2019) (35)	4C Study	3.8	Asia	55,671	2,064	23,571	1,774	12,697	1,467	14,554	2,758	RR	Adjusted for age, sex, BMI, family history of diabetes, smoking, drinking, education status, physical activity, SBP, HDL, LDL, and triglycerides.
Unjali (2019) (36)	MASALA Study	5	North America	268	6	81	7	89	17	44	13	–	–

NFG+NGT, normal fasting glucose combined with normal glucose tolerance; I-IFG, isolated impaired fasting glucose; I-IGT, isolated impaired glucose tolerance; IGT+IFG, impaired fasting glucose combined with impaired glucose tolerance; T2DM, type 2 diabetes.

### Quality Assessment

The Newcastle-Ottawa quality assessment scale was used for quality assessment of the included studies. According to the quality assessment criteria, the results of quality assessment showed that the mean score across included studies was 7.2, 13 studies were graded as good quality and 3 studies as fair (see **Supplementary Materials 2**, which illustrates the risk of bias).

### Risk of Progression to Type 2 Diabetes in the Various Prediabetic Groups

We separately described the risk of progression to type 2 diabetes for the various prediabetic groups according to two different diagnostic criteria. Random-effect models were used for analyses due to the high *I^2^* in most comparison.

#### A. I-IGT (IGT+NFG) Versus IGT+IFG Population

According to the plasma glucose level of prediabetes given by the WHO 1999 and ADA 2003 (5, 6), the impaired glucose tolerance included I-IGT (IGT+NFG) and IGT+IFG groups.

##### a. WHO 1999 Plasma Glucose Criteria

**Figure 2:**
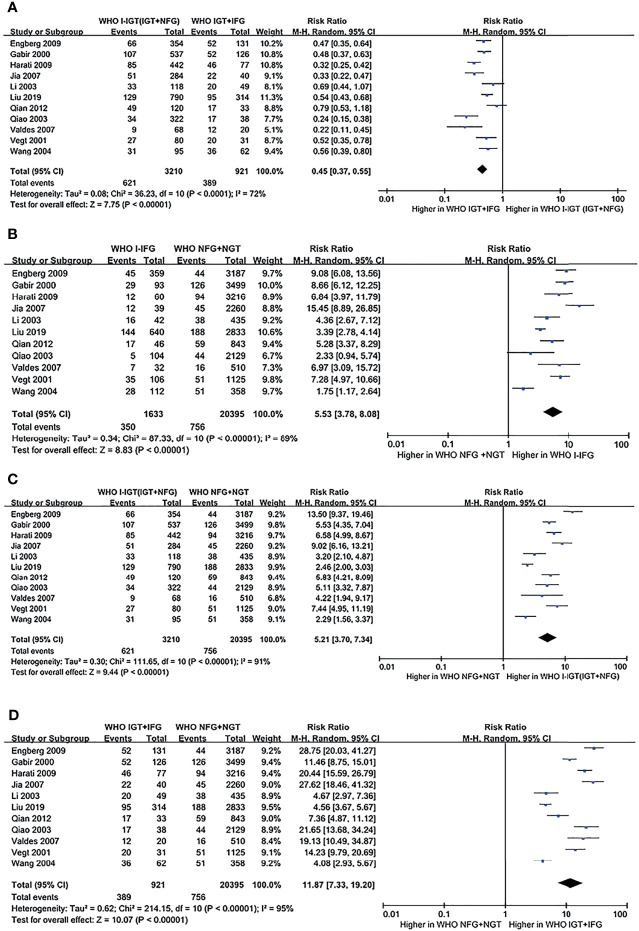
Forest plots comparing the risk of progression to type 2 diabetes between groups according to the WHO 1999 classification criteria. **(A)** The risk of progression to type 2 diabetes was higher in the WHO IGT+IFG group than in the WHO I-IGT (IGT+NFG) group. **(B)** The risk of progression to type 2 diabetes was higher in the WHO I-IFG groups than in the WHO NFG+NGT group. **(C)** The risk of progression to type 2 diabetes was higher in the WHO I-IGT (IGT+NFG) than in the WHO NFG+NGT group. **(D)** The risk of progression to type 2 diabetes was higher in the WHO IGT+IFG than in the WHO NFG+NGT group. 95% CI, 95% confidence interval; I-IGT, isolated impaired glucose tolerance; IGT+IFG, impaired fasting glucose combined with impaired glucose tolerance; I-IFG, isolated impaired fasting glucose; NFG+NGT, normal fasting glucose combined with normal glucose tolerance.

According to the WHO 1999 plasma glucose criteria, there were 3,210 individuals in the WHO I-IGT (IGT+NFG) group and 921 individuals in the WHO IGT+IFG group. The meta-analysis showed that the subsequent incidence of type 2 diabetes was lower in the WHO I-IGT (IGT+NFG) group than in the WHO IGT+IFG group (RR [95% CI] of 0.45 [0.37, 0.55]) (see **Figure 2A**).

##### b. ADA 2003 Plasma Glucose Criteria

**Figure 3:**
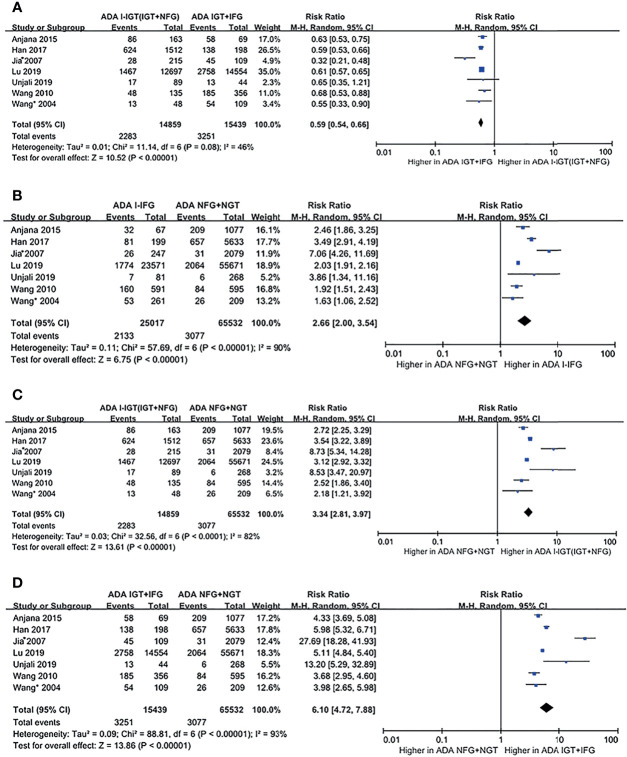
Forest plots comparing the risk of progression to type 2 diabetes between groups according to the ADA 2003 classification criteria. **(A)** The risk of progression to type 2 diabetes was higher in the ADA IGT+IFG group than in the ADA I-IGT (IGT+NFG) group. **(B)** The risk of progression to type 2 diabetes was higher in the ADA I-IFG group than in the ADA NFG+NGT group. **(C)** The risk of progression to type 2 diabetes was higher in the ADA I-IGT (IGT+NFG) group than in the ADA NFG+NGT group. **(D)** The risk of progression to type 2 diabetes was higher in the ADA IGT+IFG groups than in the ADA NFG+NGT group. 95% CI, 95% confidence interval; I-IGT, isolated impaired glucose tolerance; IGT+IFG, impaired fasting glucose combined with impaired glucose tolerance; I-IFG, isolated impaired fasting glucose; NFG+NGT, normal fasting glucose combined with normal glucose tolerance.

According to the ADA 2003 plasma glucose criteria, there were 14,859 individuals in the ADA I-IGT (IGT+NFG) group and 15,439 individuals in the ADA IGT+IFG group. The meta-analysis showed that the subsequent incidence of type 2 diabetes was lower in the ADA I-IGT (IGT+NFG) group than in the ADA IGT+IFG group (RR [95% CI] of 0.59 [0.54, 0.66]) (see **Figure 3A**).

#### B. Prediabetic Population Versus the Normoglycemic Population

##### a. WHO 1999 Plasma Glucose Criteria

According to the WHO 1999 criteria, a total of 11 articles describing 26,159 individuals were included in the analysis. There were 1,633 individuals in the WHO I-IFG group, 3,210 individuals in the WHO I-IGT (IGT+NFG) group, and 921 individuals in the WHO IGT+IFG group. The meta-analysis showed that the subsequent incidence of type 2 diabetes was higher in the WHO I-IFG, WHO I-IGT (IGT+NFG), and WHO IGT+IFG groups than in the normal glucose tolerance group (WHO NFG+NGT group) (RR [95% CI] of 5.53 [3.78, 8.08], 5.21 [3.70, 7.34], and 11.87 [7.33, 19.20]), respectively (see **Figures 2B** **–D**).

##### b. ADA 2003 Plasma Glucose Criteria

According to the ADA 2003 criteria, a total of 7 articles describing 120,847 individuals were included in the analysis. There were 25,017 individuals in the ADA I-IFG group, 14,859 individuals in the ADA I-IGT (IGT+NFG) group, and 15,439 individuals in the ADA IGT+IFG group. The meta-analysis showed that the incidence of type 2 diabetes was higher in the ADA I-IFG, ADA I-IGT (IGT+NFG), and ADA IGT+IFG groups than in the ADA NFG+NGT group (RR [95% CI] of 2.66 [2.00, 3.54], 3.34 [2.81, 3.97], and 6.10 [4.72, 7.88]), respectively (see **Figures 3B** **–D**).

### Subgroup Analysis

Subgroup analyses were conducted based on duration and region for different comparisons, classified by WHO and ADA criteria; results show no significant effect between any subgroups, and results in subgroup analysis coincide with results above (**Figures 4**, **5**; **Supplementary Figures S1–S6**).

**Figure 4:**
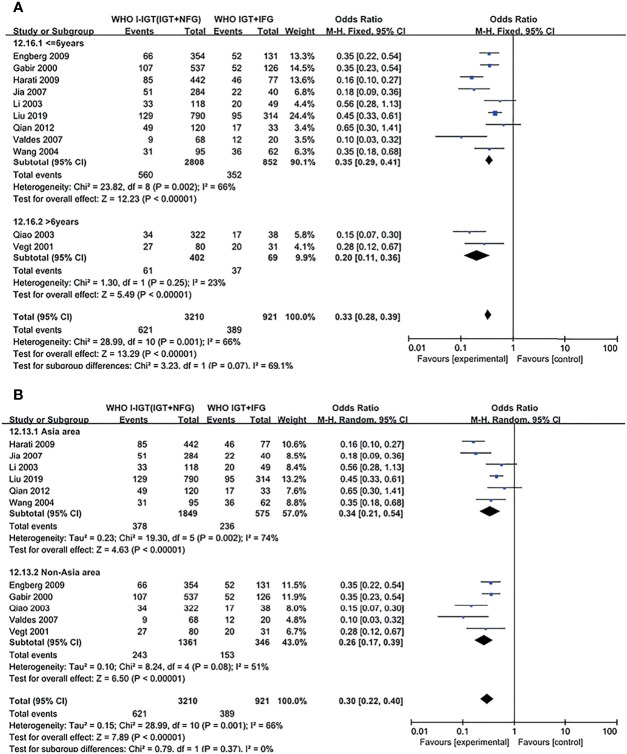
Subgroup analysis was performed according to follow-up duration **(A)** and study location **(B)** in WHO 1999 groups. Subgroup analysis showed that it did not affect the final outcome. 95% CI, 95% confidence interval; I-IGT, isolated impaired glucose tolerance; IGT+IFG, impaired fasting glucose combined with impaired glucose tolerance.

**Figure 5:**
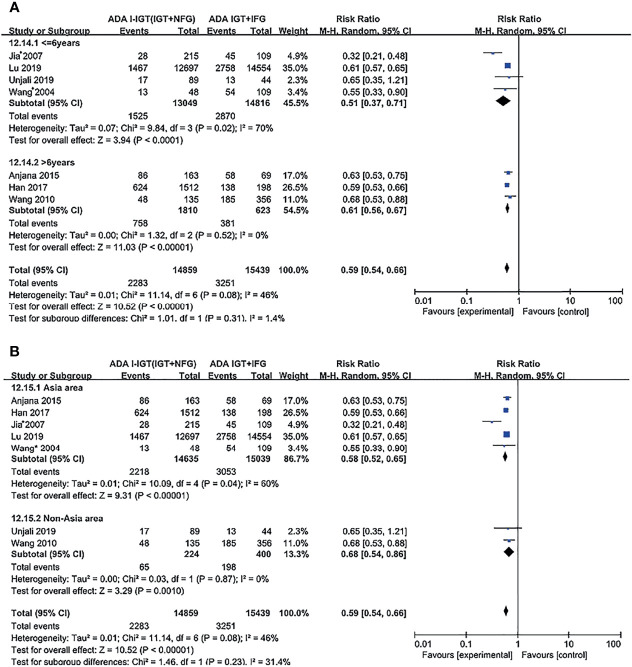
Subgroup analysis was performed according to follow-up duration **(A)** and study location **(B)** in the ADA 2003 groups. Subgroup analysis showed that it did not affect the final outcome. 95% CI, 95% confidence interval; I-IGT, isolated impaired glucose tolerance; IGT+IFG, impaired fasting glucose combined with impaired glucose tolerance.

### Publication Bias

**Figure 6:**
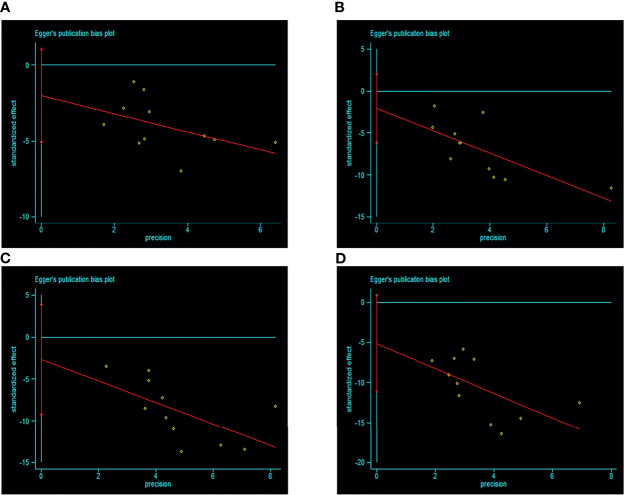
Egger’s funnel plot of included studies for publication bias in WHO 1999 subgroup [**(A)** IGT+IFG group VS I-IGT (IGT+NFG) group. **(B)** I-IFG groups VS NFG+NGT group. **(C)** I-IGT (IGT+NFG) VS NFG+NGT group. **(D)** IGT+IFG VS NFG+NGT group]. Egger’s test result showed that there was no publication bias (p > 0.05). 95% CI, 95% confidence interval; I-IGT, isolated impaired glucose tolerance; IGT+IFG, impaired fasting glucose combined with impaired glucose tolerance; I-IFG, isolated impaired fasting glucose; NFG+NGT, normal fasting glucose combined with normal glucose tolerance.

There were 11 studies included according to the WHO 1999 criteria; visual inspection of funnel plots showed symmetry in this subgroup (see **Figure 6**). Egger’s test result showed that there was no publication bias (*p* > 0.05). Only 7 studies were included according to the ADA 2003 criteria, and no publication bias analysis was done because of the small number of studies.

### Sensitivity Analysis

Sensitivity analysis using alternative effect measures did not show important changes in pooled effects (see **Supplementary Figure S7**).

## Discussion

Diabetes is usually preceded by a condition known as prediabetes. The WHO 1999 diagnostic criteria for impaired glucose tolerance do not distinguish normal fasting glucose or impaired fasting glucose when the 2-h plasma glucose during an OGTT is ≥7.8 mmol/L (140 mg/dl) and <11.1 mmol/L (200 mg/dl), but the ADA 2003 criteria distinguish these subgroups (I-IGT (IGT+NFG) and IGT+IFG). Thus, there is some controversy as to whether the IGT should be subdivided into I-IGT (IGT+NFG) and IGT+IFG groups, and the risk of progression to type 2 diabetes for each of these subgroups is not well characterized. Our meta-analysis of data from 16 studies compared the risk of progression to type 2 diabetes for individuals in the I-IGT (IGT+NFG) and IGT+IFG groups, and between the I-IFG, I-IGT (IGT+NFG), and IGT+IFG subgroups and normoglycemic subjects. We found that the subsequent incidence of type 2 diabetes was lower in the I-IGT (IGT+NFG) group than in the IGT+IFG group when either the WHO 1999 and ADA 2003 criteria were applied. We also found that the subsequent incidence of type 2 diabetes was higher in the I-IFG, I-IGT (IGT+NFG), and IGT+IFG groups than in the normoglycemic group when either set of criteria were used. Thus, the subsequent incidence of type 2 diabetes in the IGT+IFG group was higher than that of the I-IGT (IGT+NFG) group, and the incidence of diabetes in the IGT+IFG group was the highest in the prediabetic population.

Our study has some limitations that are common to meta-analyses. First, statistical heterogeneity was relatively high, which is an inherent flaw in epidemiologic studies and meta-analyses, especially in observational studies. Because of the lack of available data from the included studies, we could hardly conduct a meta-regression analysis due to possible factors were not taken into account, such as variations possibly associate with age, composition of gender, or other socioenvironmental factors. Additionally, due to the limitations of original large-scale epidemiological investigations, it is not possible to completely distinguish T2DM from type 1 diabetes mellitus (T1DM) or latent autoimmune diabetes in adults (LADA), although T2DM occurs in more than 90% of the total diabetic population (1), which may also contribute to the relatively high statistical heterogeneity. Second, only 7 studies were included in the ADA 2003 group and were unreliable for publication bias, therefore the significant differences of the risk of progression to type 2 diabetes between prediabetes subgroups and normoglycemic group might be overestimated. Although high-quality studies are usually published in English, there may be other high-quality studies published in other languages that have not been included.

Despite the above limitations, our study has clear advantages. First, we compared the progression with type 2 diabetes in the I-IGT (IGT+NFG) group with the IGT+IFG group. This comparison permits us to answer the important practical question of whether the impaired glucose tolerance should be divided into I-IGT (IGT+NFG) and IGT+IFG subgroups. Second, we clearly distinguish I-IFG, I-IGT (IGT+NFG), and IGT+IFG subgroups of prediabetes, while many studies that report the prognosis, conversion, or interventions in prediabetes do not clearly distinguish these three types of subjects. Third, the follow-up period used in the included studies was long and the median duration was 5.6 years. Fourth, we repartitioned the included studies into two categories according to both WHO 1999 and ADA 2003 plasma glucose criteria for analysis. This strategy should reduce the confounding effect of the use of different criteria among the included studies. Finally, to our knowledge, this is the first meta-analysis to be conducted on this topic in recent years. Although a meta-analysis of progression to type 2 diabetes in I-IFG, I-IGT (IGT+NFG), and IGT+IFG prediabetic groups was published in 2005, there were only three studies included (37), while other studies focused on the complications of the prediabetes (38–40). Therefore, our findings represent an important contribution to the epidemiologic study of prediabetes.

We found that the subsequent incidence of type 2 diabetes in the I-IGT (IGT+NFG) group was lower than that of the IGT+IFG group. Therefore, we believe that IGT with or without IFG is important for the progression to type 2 diabetes, and impaired glucose tolerance category should be divided into I-IGT (IGT+NFG) and IGT+IFG groups and the use of impaired glucose tolerance as a category should be abandoned. In addition, we found that the subsequent incidence of type 2 diabetes was different among the I-IFG, I-IGT (IGT+NFG), and IGT+IFG groups, and that the incidence of type 2 diabetes was highest in the IGT+IFG group. Results in subgroup analysis based on duration and region coincide with results above. However, the definition and classification of prediabetes in the newly released WHO 2006/ADA 2019 standards of care has no difference from the WHO1999/ADA 2003 criteria. We believe that the I-IFG, I-IGT (IGT+NFG), and IGT+IFG categories of prediabetes should be used in future epidemiological studies of prediabetes. Moreover, by plasma glucose level, the combination of IFG and I-IGT (IGT+NFG) was the strongest predictor of incident diabetes in the prediabetic population, implying that both fasting plasma glucose and 2-h plasma glucose during an OGTT should be assessed to classify prediabetes.

In addition to the combination of IFG and I-IGT (IGT+NFG) as important risk factors for the progression of type 2 diabetes, many other risk factors are associated with type 2 diabetes. Vegt et al. observed that the waist hip rate (WHR) was an important predictor for progression to type 2 diabetes (22), and Harati’s study showed that the obesity and hypertriglyceridemia were also risk factors of progression to incident type 2 diabetes (28). Therefore, the establishment of a comprehensive and practical risk model of type 2 diabetes has great significance in clinical practice. Identification of those who may be at high risk of developing diabetes is usually the first and important step for the prevention of type 2 diabetes. Based on the risk of developing type 2 diabetes, different interventions and treatments in populations could contribute to the prevention, treatment, and management of type 2 diabetes.

In conclusion, the subsequent incidence of type 2 diabetes in the I-IGT (IGT+NFG) group was lower than that of the IGT+IFG group. The subsequent incidence of type 2 diabetes in the IGT+IFG group was the highest among all the prediabetic population (I-IFG, I-IGT (IGT+NFG), and IGT+IFG groups), and it was higher in the I-IFG, I-IGT (IGT+NFG), and IGT+IFG groups than in the normoglycemic group. Thus, the I-IGT (IGT+NFG) and IGT+IFG groups have different risks of progression to type 2 diabetes, and therefore the established WHO criteria defining IGT should be revised to define I-IGT (IGT+NFG) and IGT+IFG subgroups. Both fasting plasma glucose and 2-h plasma glucose during an OGTT should be evaluated to classify prediabetes, to distinguish I-IFG, I-IGT (IGT+NFG), and IGT+IFG subgroups. We suggest that prediabetes should be classified as I-IFG, I-IGT (IGT+NFG), and IGT+IFG clinically and in epidemiologic studies.

## Data Availability Statement

The raw data supporting the conclusions of this article will be made available by the authors, without undue reservation.

## Author Contributions

YL and NT participated in the study design. YL and JL participated in data acquisition, analyzing data, and preparation of the manuscript. YL, JL, YW, and XZ participated in data interpretation. All authors provided critical revision and editing of the manuscript. All authors listed have made a substantial, direct, and intellectual contribution to the work and approved it for publication.

## Funding

This work was supported by the Sichuan Provincial Science and Technology Foundation for Major Project (2015SZ0228), the Program of National Natural Science Foundation of China (82070846), and Program for Overseas High-Level Talent Introduction of Sichuan Province of China (2021JDGD0038). We thank Mark Cleasby, PhD, from Liwen Bianji, Edanz Group China (www.liwenbianji.cn/ac), for editing the English text of a draft of this manuscript.

## Conflict of Interest

The authors declare that the research was conducted in the absence of any commercial or financial relationships that could be construed as a potential conflict of interest.

## Publisher’s Note

All claims expressed in this article are solely those of the authors and do not necessarily represent those of their affiliated organizations, or those of the publisher, the editors and the reviewers. Any product that may be evaluated in this article, or claim that may be made by its manufacturer, is not guaranteed or endorsed by the publisher.
