# Supplementary material for: Evidence From a Systematic Review and Meta-Analysis: Classical Impaired Glucose Tolerance Should Be Divided Into Subgroups of Isolated Impaired Glucose Tolerance and Impaired Glucose Tolerance Combined With Impaired Fasting Glucose, According to the Risk of Progression to Diabetes
Source: Front Endocrinol (Lausanne). 2022 Feb 18;13:835460. doi: 10.3389/fendo.2022.835460 (PMC8894674; doi:10.3389/fendo.2022.835460)
Supplement: Supplementary file 1 [file DataSheet_1.zip › Supplementary Figures S1-S7.docx]

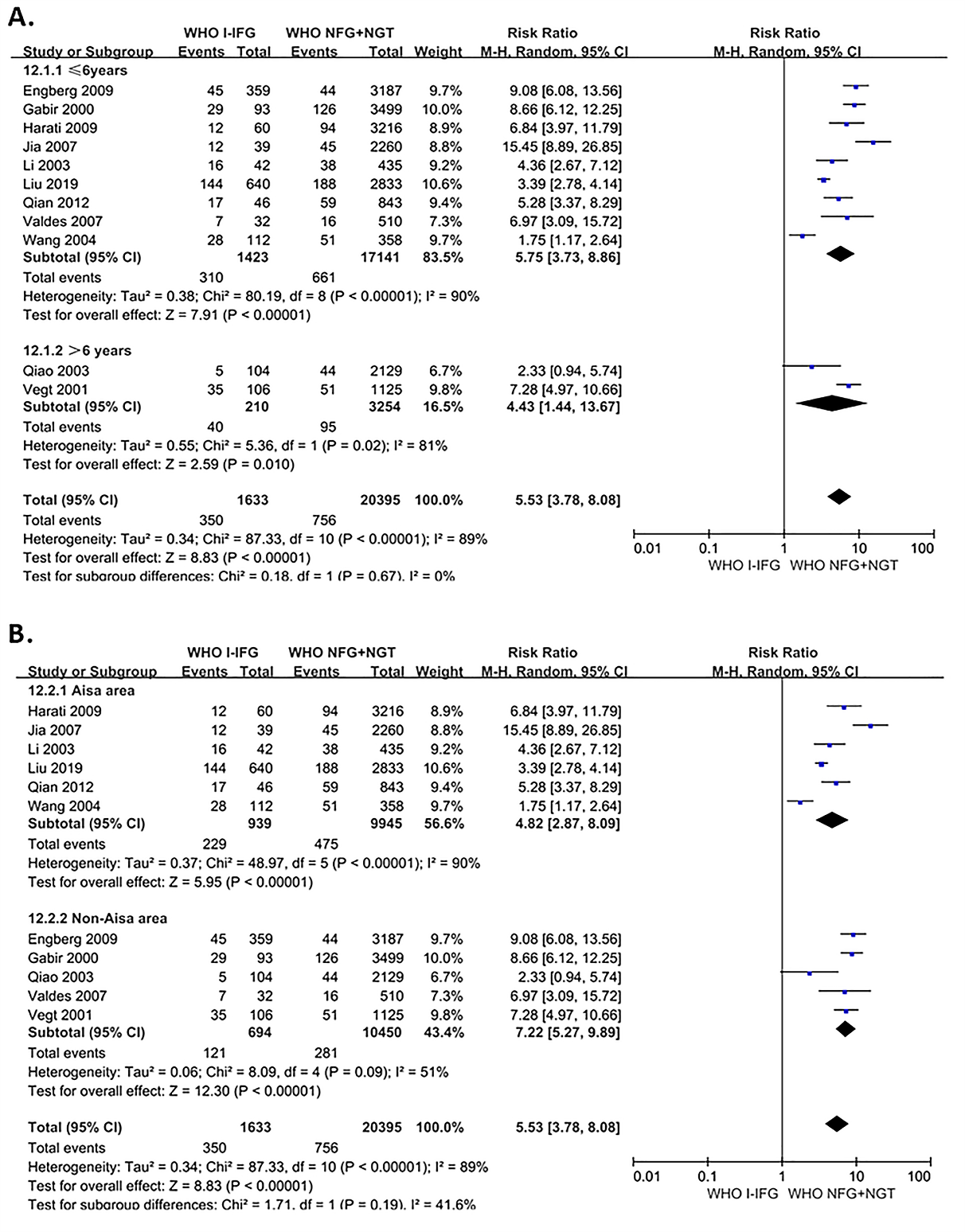


**Figure** S1: According to follow-up duration(A) and study location(B), Subgroup analysis was performed between I-IFT and NFG+NGT in WHO 1999 criteria. Subgroup analysis showed that it did not affect the final outcome.

95% CI = 95% confidence interval. I-IFG: isolated impaired fasting glucose; NFG+NGT: normal fasting glucose combined with normal glucose tolerance.


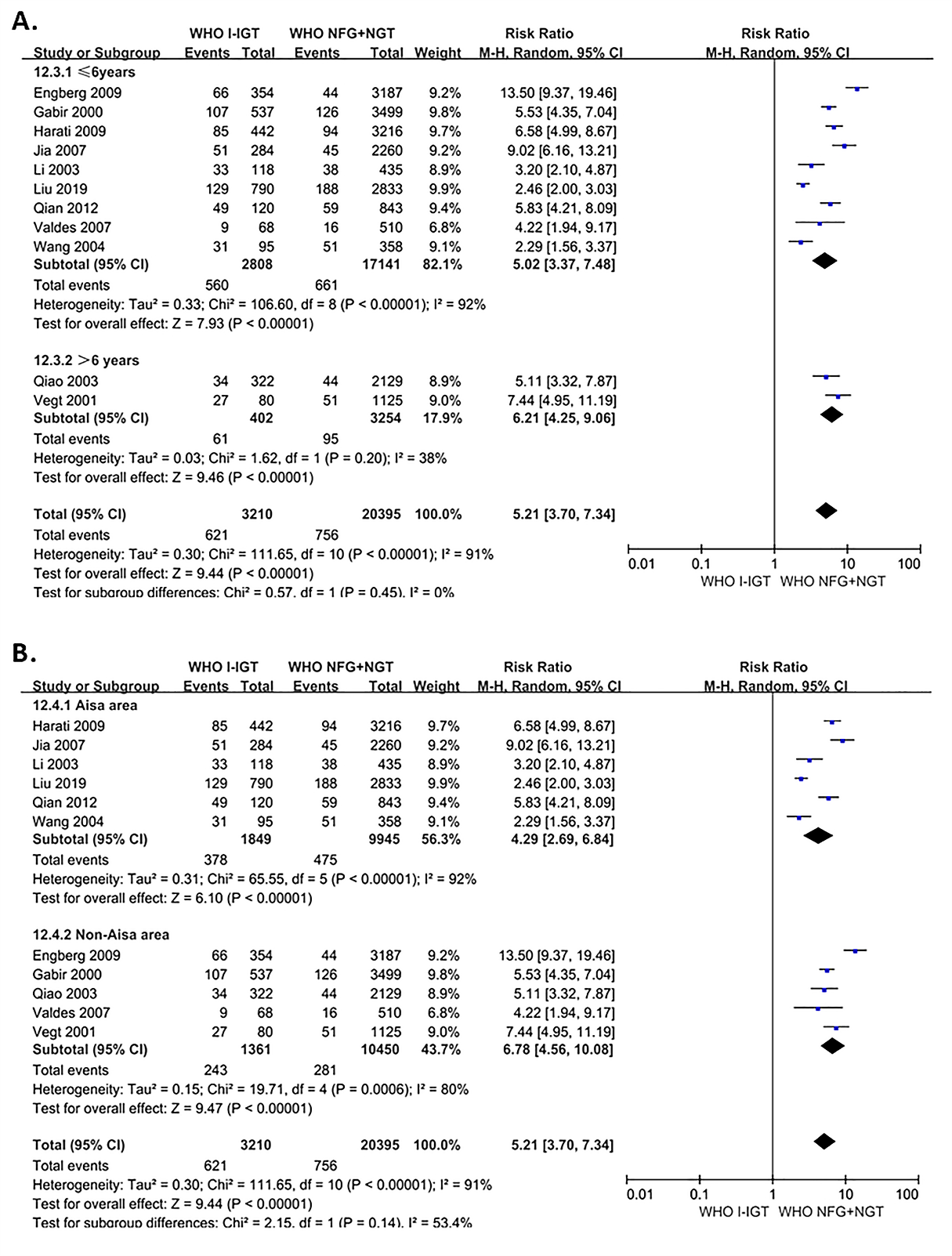


**Figure** S2: According to follow-up duration(A) and study location(B), Subgroup analysis was performed between I-IGT and NFG+NGT in WHO 1999 criteria. Subgroup analysis showed that it did not affect the final outcome.

95% CI = 95% confidence interval. I-IGT: isolated impaired glucose tolerance;

NFG+NGT: normal fasting glucose combined with normal glucose tolerance.


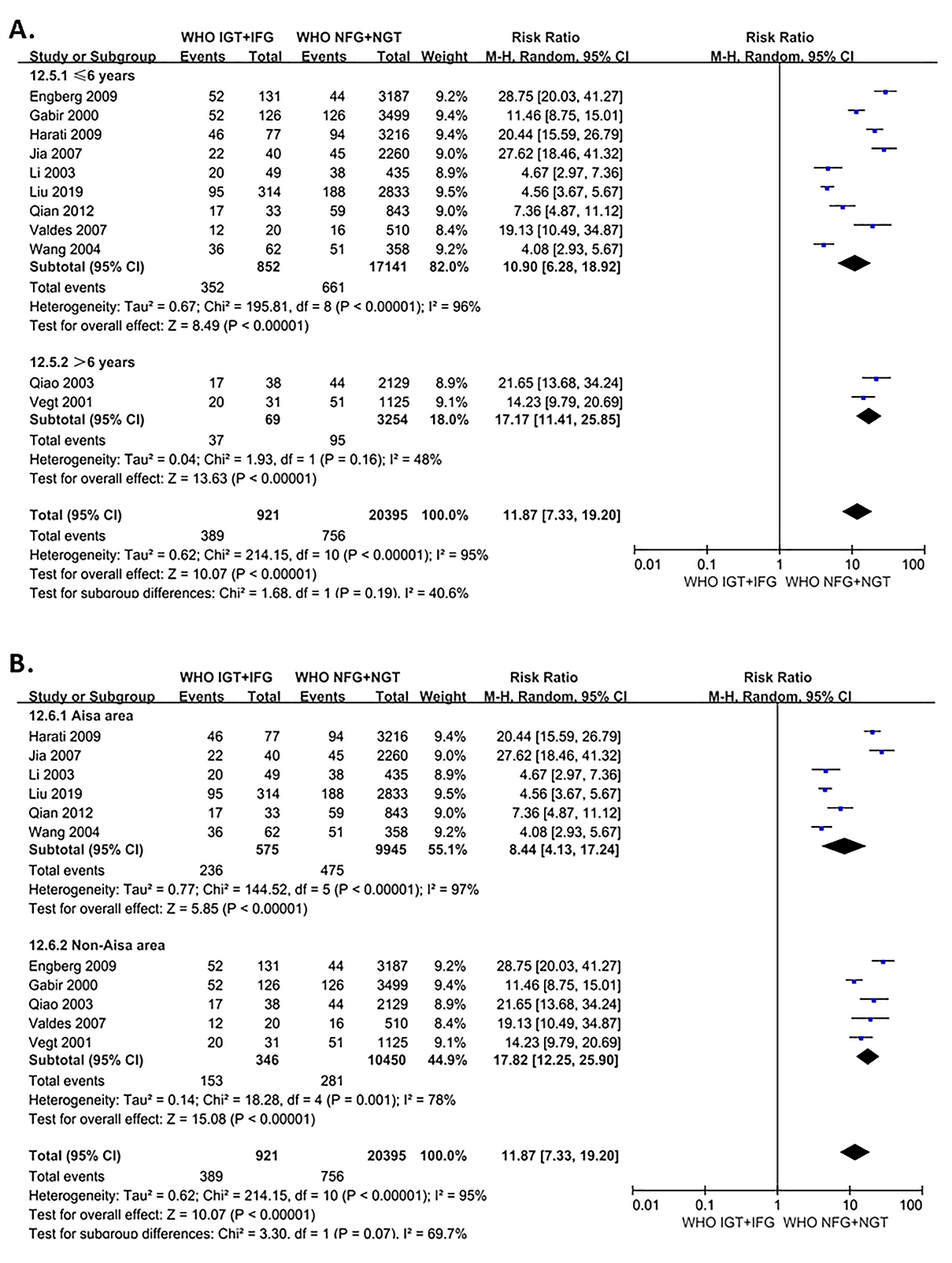


**Figure** S3: According to follow-up duration(A) and study location(B), Subgroup analysis was performed between IGT+IFG and NFG+NGT in WHO 1999 criteria. Subgroup analysis showed that it did not affect the final outcome.

95% CI = 95% confidence interval. IGT+IFG: impaired fasting glucose combined with impaired glucose tolerance; NFG+NGT: normal fasting glucose combined with normal glucose tolerance.


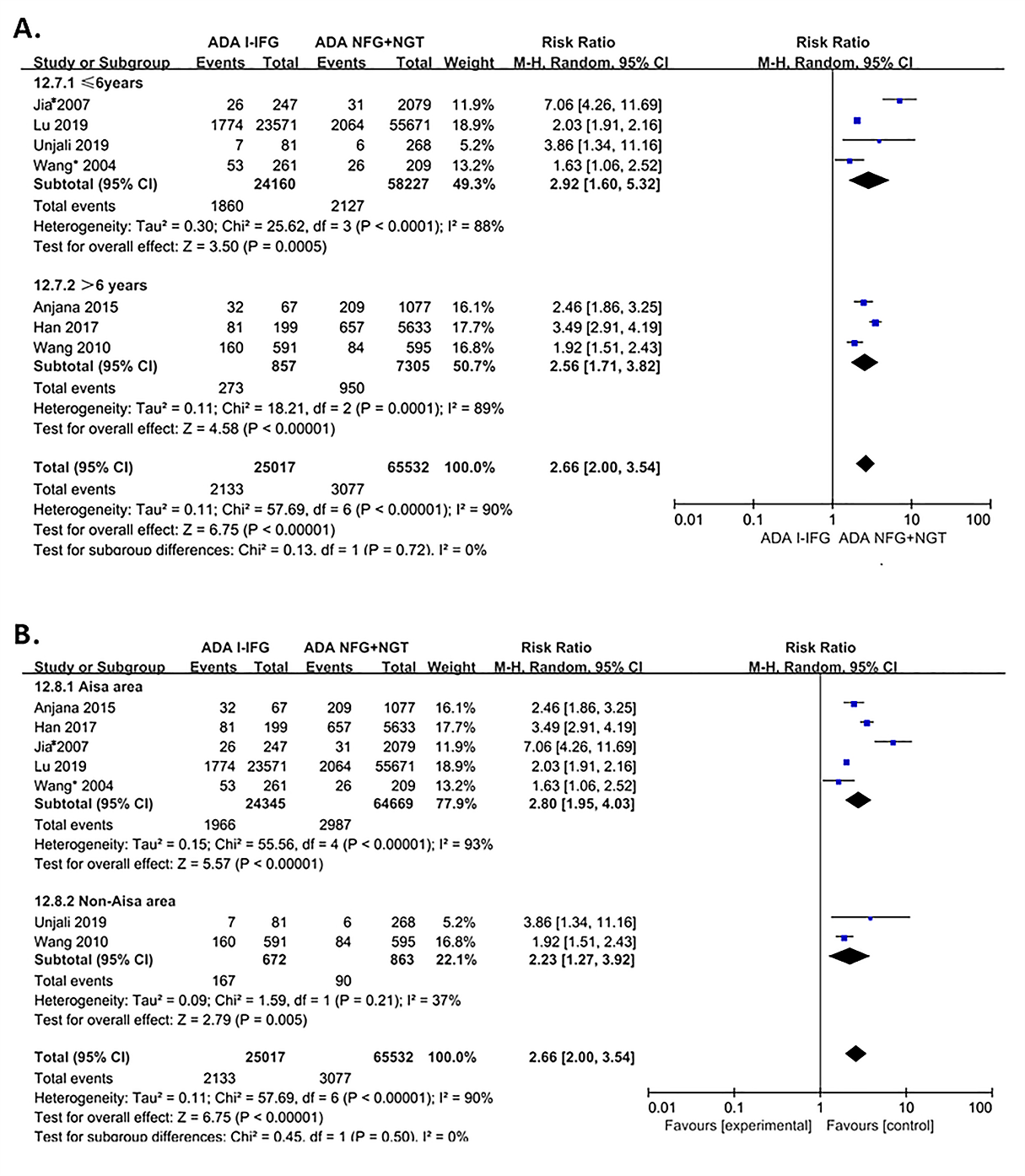


**Figure** S4: According to follow-up duration(A) and study location(B), Subgroup analysis was performed between I-IFT and NFG+NGT in ADA 2003 criteria. Subgroup analysis showed that it did not affect the final outcome.

95% CI = 95% confidence interval. I-IFG: isolated impaired fasting glucose; NFG+NGT: normal fasting glucose combined with normal glucose tolerance.


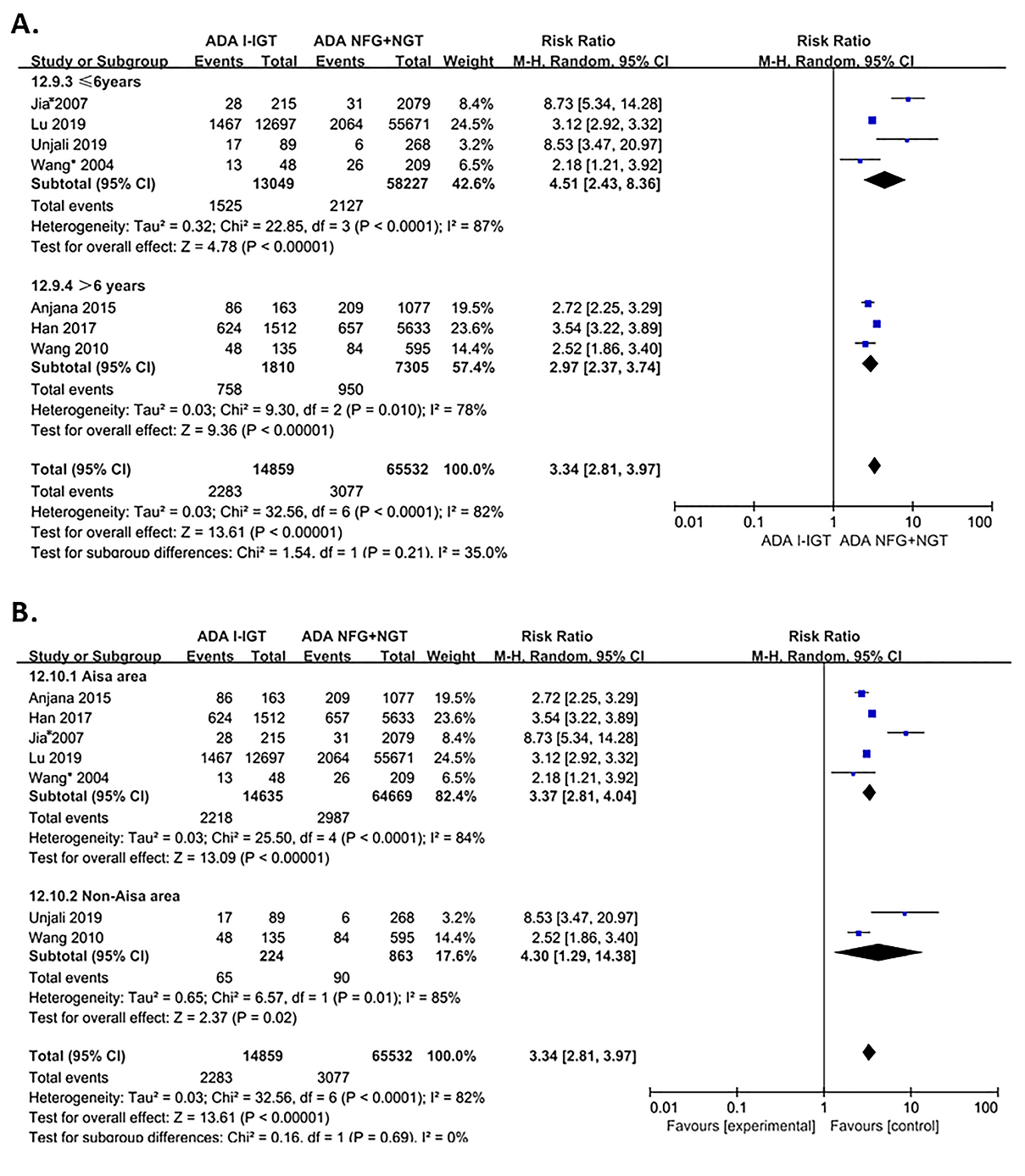


**Figure** S5: According to follow-up duration(A) and study location(B), Subgroup analysis was performed between I-IGT and NFG+NGT in ADA 2003 criteria. Subgroup analysis showed that it did not affect the final outcome.

95% CI = 95% confidence interval. I-IGT: isolated impaired glucose tolerance; NFG+NGT: normal fasting glucose combined with normal glucose tolerance.


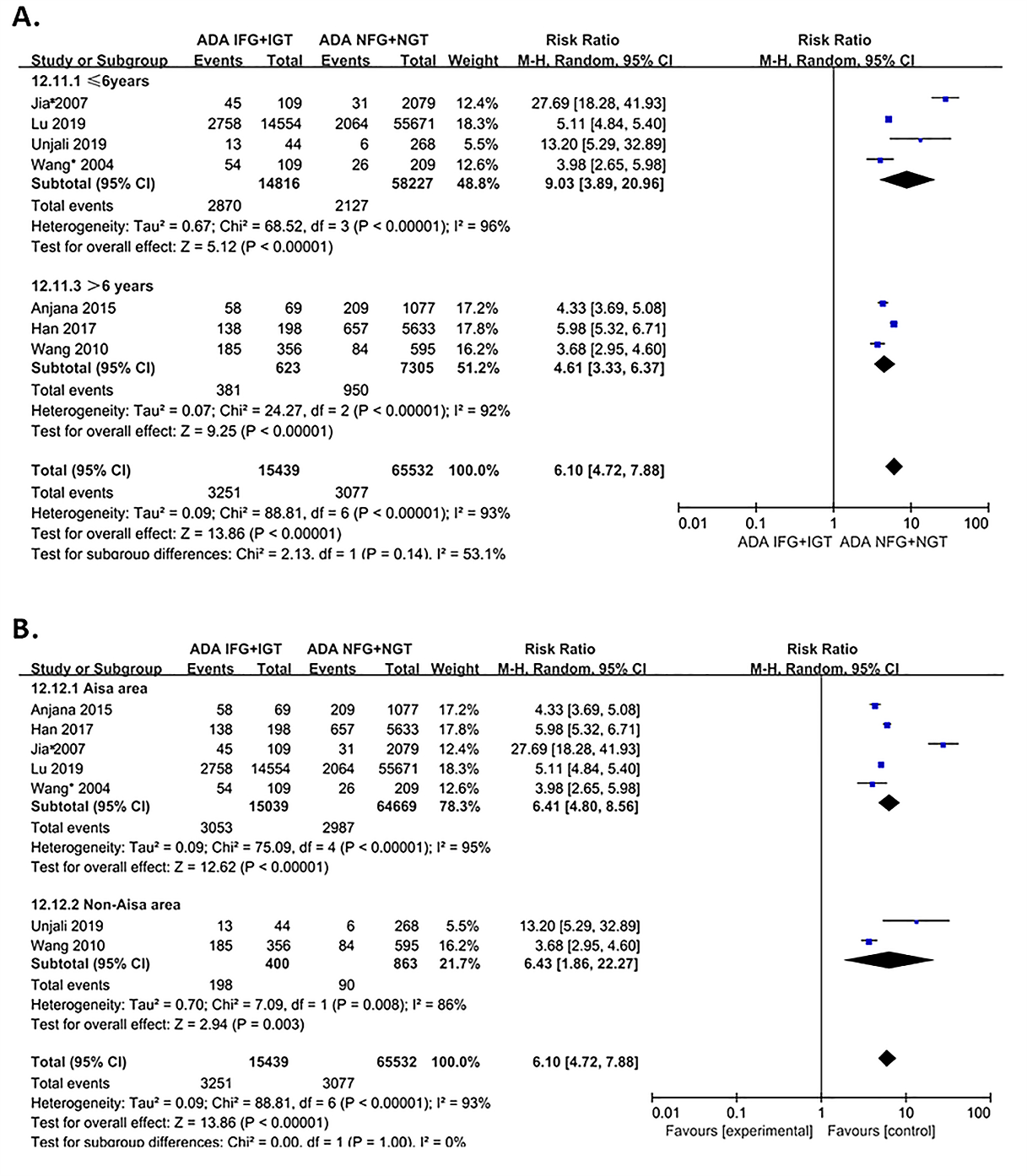


**Figure** S6: According to follow-up duration(A) and study location(B), Subgroup analysis was performed between IGT+IFG and NFG+NGT in ADA 2003 criteria. Subgroup analysis showed that it did not affect the final outcome.

95% CI = 95% confidence interval. IGT+IFG: impaired fasting glucose combined with impaired glucose tolerance; NFG+NGT: normal fasting glucose combined with normal glucose tolerance.


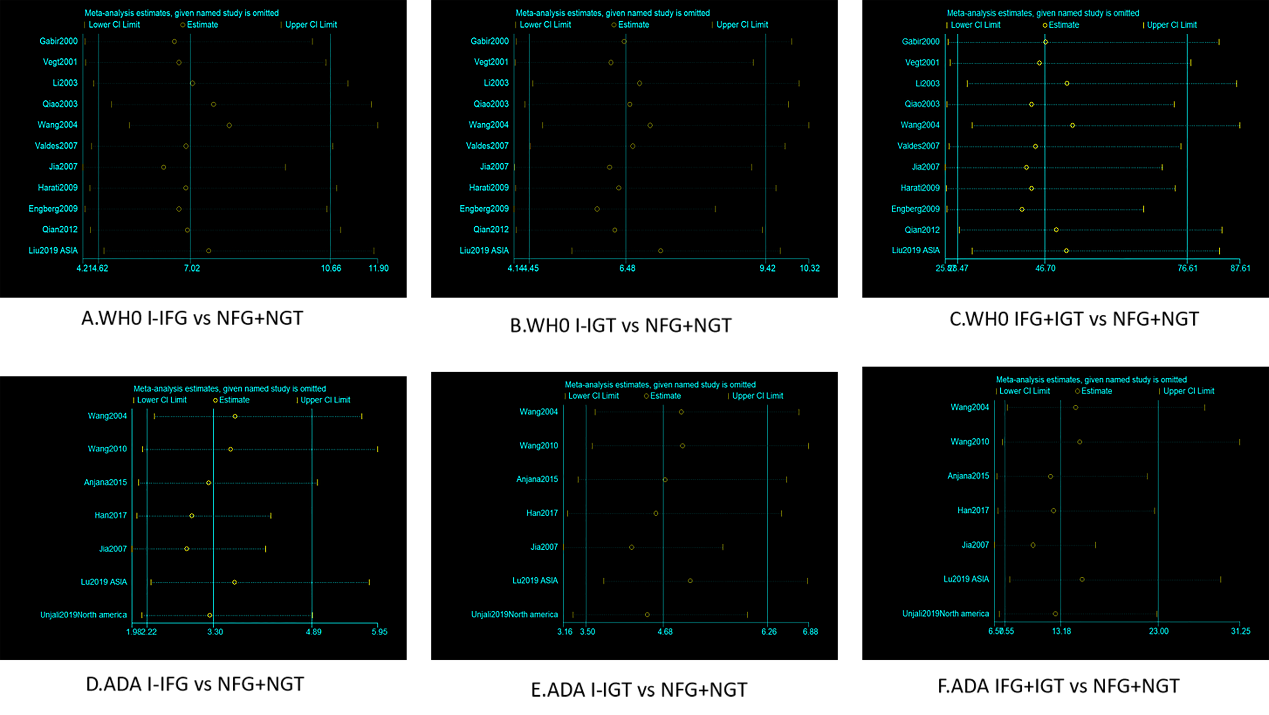


**Figure** S7: Sensitivity analysis of studies included in meta-analysis, did not show important changes in pooled effects.
