# Supplementary material for: Evidence From a Systematic Review and Meta-Analysis: Classical Impaired Glucose Tolerance Should Be Divided Into Subgroups of Isolated Impaired Glucose Tolerance and Impaired Glucose Tolerance Combined With Impaired Fasting Glucose, According to the Risk of Progression to Diabetes
Source: Front Endocrinol (Lausanne). 2022 Feb 18;13:835460. doi: 10.3389/fendo.2022.835460 (PMC8894674; doi:10.3389/fendo.2022.835460)
Supplement: Supplementary file 1 [file DataSheet_1.zip › Supplementary Material 1.docx]

**Search strategies**

Medline

1. exp Prediabetic State/
2. (igt or ifg).tw.
3. exp Glucose Intolerance/
4. (impaired glucose or impaired fasting glucose).tw.
5. 1 or 2 or 3 or 4
6. exp Prognosis/
7. (progress or follow up or prediction).tw.
8. 6 or 7
9. 5 and 8

Embase

1. exp glucose intolerance/
2. exp impaired glucose tolerance/
3. (impaired glucose or impaired fasting glucose).tw.
4. (IGT or IFG).tw.
5. prediabet:.tw.
6. pre-diabet:.tw.
7. 1 or 2 or 3 or 4 or 5 or 6
8. exp prognosis/
9. (progress or follow up or prediction).tw.
10. 8 or 9
11. 7 and 10
12. limit 11 to english language
