# Supplementary material for: Evidence From a Systematic Review and Meta-Analysis: Classical Impaired Glucose Tolerance Should Be Divided Into Subgroups of Isolated Impaired Glucose Tolerance and Impaired Glucose Tolerance Combined With Impaired Fasting Glucose, According to the Risk of Progression to Diabetes
Source: Front Endocrinol (Lausanne). 2022 Feb 18;13:835460. doi: 10.3389/fendo.2022.835460 (PMC8894674; doi:10.3389/fendo.2022.835460)
Supplement: Supplementary file 1 [file DataSheet_1.zip › Supplementary Material 2.doc]

**Supplementary Material 2. Risk of bias**

| Study | Selection | | | | | Compar-ability | Outcome | | | Overall |
| --- | --- | --- | --- | --- | --- | --- | --- | --- | --- | --- |
|  | Representativeness of the exposed cohort | | Selection of the no exposed cohort | Ascertainment of exposure | Demonstration that outcome of interest was not present at start of study | Comparabi-lity on the basis of the design or analysis | Assessment of outcome | Was follow-up long enough for outcomes to occur | Adequacy of follow up of cohorts |  |
| Gabir2000[22] | * | * | | * | * | * | * | * |  | 7 |
| Vegt2001[23] | * | * | | * | * | * | * | * | * | 8 |
| Li2003[24] |  |  | | * | * | * | * | * | * | 6 |
| Qiao2003[25] | * | * | | * | * | * | * | * |  | 7 |
| Wang2004[26] |  |  | | * | * | ** | * | * |  | 6 |
| Valdes2007[27] | * | * | | * | * | * | * | * | * | 8 |
| Jia2007[28] | * | * | | * | * | * | * | * |  | 7 |
| Harati2009[29] | * | * | | * | * | * | * | * |  | 7 |
| Engberg2009  [30] | * | * | | * | * | * | * | * | * | 8 |
| Wang2010[31] | * | * | | * | * | * | * | * |  | 7 |
| Qian2012[32] |  | * | | * | * | * | * | * |  | 6 |
| Anjana2015[33] | * | * | | * | * | * | * | * | * | 8 |
| Han2017[34]  Liu2019[35]  Lu2019[36]  Unjali2019[37] | *  *  *  * | *  *  *  * | | *  *  *  * | *  *  *  * | *  *  *  * | *  *  *  * | *  *  *  * | *  * | 7  8  8  7 |
